# Predicting Profile Soil Properties with Reflectance Spectra via Bayesian Covariate-Assisted External Parameter Orthogonalization

**DOI:** 10.3390/s18113869

**Published:** 2018-11-10

**Authors:** Kristen S. Veum, Paul A. Parker, Kenneth A. Sudduth, Scott H. Holan

**Affiliations:** 1 USDA-ARS Cropping Systems and Water Quality Research Unit, Columbia, MO 65211, USA; ken.sudduth@ars.usda.gov; 2 Department of Statistics, University of Missouri, Columbia, MO 65211, USA; papnp7@mail.missouri.edu (P.A.P.); holans@missouri.edu (S.H.H.)

**Keywords:** Bayesian Lasso, diffuse reflectance spectroscopy, external parameter orthogonalization, partial least squares regression, profile soil properties, proximal soil sensing, soil carbon, soil texture

## Abstract

In situ, diffuse reflectance spectroscopy (DRS) profile soil sensors have the potential to provide both rapid and high-resolution prediction of multiple soil properties for precision agriculture, soil health assessment, and other applications related to environmental protection and agronomic sustainability. However, the effects of soil moisture, other environmental factors, and artefacts of the in-field spectral data collection process often hamper the utility of in situ DRS data. Various processing and modeling techniques have been developed to overcome these challenges, including external parameter orthogonalization (EPO) transformation of the spectra. In addition, Bayesian modeling approaches may improve prediction over traditional partial least squares (PLS) regression. The objectives of this study were to predict soil organic carbon (SOC), total nitrogen (TN), and texture fractions using a large, regional dataset of in situ profile DRS spectra and compare the performance of (1) traditional PLS analysis, (2) PLS on EPO-transformed spectra (PLS-EPO), (3) PLS-EPO with the Bayesian Lasso (PLS-EPO-BL), and (4) covariate-assisted PLS-EPO-BL models. In this study, soil cores and in situ profile DRS spectrometer scans were obtained to ~1 m depth from 22 fields across Missouri and Indiana, USA. In the laboratory, soil cores were split by horizon, air-dried, and sieved (<2 mm) for a total of 708 samples. Soil properties were measured and DRS spectra were collected on these air-dried soil samples. The data were randomly split into training (n = 308), testing (n = 200), and EPO calibration (n = 200) sets, and soil textural class was used as the categorical covariate in the Bayesian models. Model performance was evaluated using the root mean square error of prediction (RMSEP). For the prediction of soil properties using a model trained on dry spectra and tested on field moist spectra, the PLS-EPO transformation dramatically improved model performance relative to PLS alone, reducing RMSEP by 66% and 53% for SOC and TN, respectively, and by 76%, 91%, and 87% for clay, silt, and sand, respectively. The addition of the Bayesian Lasso further reduced RMSEP by 4–11% across soil properties, and the categorical covariate reduced RMSEP by another 2–9%. Overall, this study illustrates the strength of the combination of EPO spectral transformation paired with Bayesian modeling techniques to overcome environmental factors and in-field data collection artefacts when using in situ DRS data, and highlights the potential for in-field DRS spectroscopy as a tool for rapid, high-resolution prediction of soil properties.

## 1. Introduction

On-the-go diffuse reflectance spectroscopy (DRS) sensors in the visible (VIS; 400–700 nm) and near-infrared (NIR; 700–2500 nm) range have the potential to provide high-resolution spatial data quickly at low cost. Soil properties are known to exhibit high spatial variability across landscapes and with depth, and soil profile characteristics are important factors in understanding hydrology, soil productivity, and other soil functions. Therefore, the ability to reliably predict soil profile properties in the field would increase the effectiveness of site-specific agriculture, be beneficial for sustainable agricultural management, and have many applications in soil mapping and monitoring. Prediction of multiple soil properties using DRS has been successful on air-dried surface samples [1,2,3], air-dried whole-profile soil samples [4], and moist samples [1,3,5] scanned in the laboratory. Some studies have compared predictions obtained at different soil moisture levels, with some reporting better results with dry soil [3,6] and others reporting better results with moist soil [7,8]. Moreover, in a field setting, spectra are sensitive to other environmental conditions (e.g., temperature and soil structure) along with soil moisture, decreasing prediction accuracy and the utility of spectra collected in the field [3,5,9,10,11,12].

Various techniques have been applied to account for moisture and other environmental factors to improve model performance, including external parameter orthogonalization (EPO), direct standardization (DS), and global moisture modelling (GMM) [13]. The EPO algorithm removes variation due to external factors by projecting the soil spectra orthogonal to the space of unwanted variation [14]. Studies have successfully applied EPO for estimation of soil properties, including soil carbon [9,15] and clay content [15,16]. Alternatively, the DS approach derives a transfer matrix to characterize differences between corresponding field and laboratory spectra, and has successfully been used to predict soil organic matter using a portable spectrometer [12]. With the GMM technique, a secondary variable with a relationship to the primary variable is intentionally manipulated, resulting in a more robust calibration model [17]. This approach, akin to spiking, has been applied to datasets that span large geographical regions or use combined spectral libraries to estimate soil carbon and clay content [18,19]. The ultimate goal is to leverage existing libraries consisting of spectra collected on dry, laboratory-processed soils for prediction of soil properties from spectra collected in situ under variable environmental conditions.

The effectiveness of DRS spectra in modeling soil properties also varies depending on spectral preprocessing techniques, calibration and modeling techniques, the specific soil properties of interest, and the size and distribution of the dataset [5,20,21]. Common approaches to model calibration include partial least squares (PLS) regression [22,23] and principal components regression [2,22,24]. Alternative techniques, such as Bayesian modeling approaches for PLS, have not been extensively applied to DRS spectral datasets for profile soil property prediction, and warrant exploration. For more detailed information, comprehensive reviews of DRS applications and tools for proximal soil sensing have previously been published [20,24,25,26].

Despite these challenges, DRS sensors have been successfully applied in field settings [11,27,28]. For in situ profile data collection to a depth of 1 m, a commercial instrument, the Veris P4000 VIS-NIR-EC-force probe (Veris Technologies, Salina, Kans.), has recently become available [29]. This instrument has demonstrated success in soil C estimation across multiple fields in Kansas, USA [27]; however, in comparison to DRS spectra collected in the laboratory on dry soil, the Veris P4000 spectrometer was less accurate in soil C estimation in the 1302–2202 nm range [30]. Only a few studies on in situ profile DRS spectroscopy applications with the P4000 have been reported [31,32], and there is a need to continue to evaluate the performance of the instrument under different field conditions, across multiple soil types, and for additional soil properties. 

The objectives of this study were to compare predictions of profile soil properties, including SOC, TN, clay, silt, and sand content, using DRS spectra from: (1) in situ profile Veris P4000 DRS spectrometer scans under field moist conditions, and (2) laboratory Veris P4000 DRS spectrometer scans of air-dried soil using the following modeling approaches: (1) PLS regression, (2) EPO transformation of spectra followed by PLS regression (EPO-PLS), (3) EPO-PLS with the Bayesian Lasso (EPO-PLS-BL), and (4) adding a categorical covariate to the model (EPO-PLS-BL-C).

## 2. Materials and Methods

### 2.1. Site Characteristics

Soil sampling locations were selected in 22 fields across five major land resource areas (MLRAs) in Missouri and Indiana, USA, with varying soil type and textural classes (Table 1). Locations within each field were identified to encompass the range of landscape variation. Across this region, the principal crops are corn, soybean, cotton, feed grains, and hay, and the dominant soil orders are alfisols, mollisols, and entisols characterized by smectitic clay mineralogy. The major resource concerns are water erosion, surface water quality, loss of soil organic matter, and productivity of soils. Three fields were located in the Heavy Till Plain Area (MLRA 109), an area of rolling hills with upland divides covered by loess, underlain by glacial drift, and characterized by high clay content. Five fields were located in the Central Claypan Area (MLRA 113) on nearly level, old till plains covered with loess, underlain by glacial drift, and characterized by high clay content and complex runoff and infiltration phenomena. Six fields were located in the Northern Indiana Drift Plain Area (MLRA 98), a broad glaciated plain that is deeply mantled by till and outwash. The land surface is nearly level and the soils are of mixed mineralogy. Three fields were located in the Central Mississippi Valley Wooded Slopes Area (MLRA 115B) in the Missouri River flood plain, where glacial outwash, alluvium, and sandy eolian materials were deposited on stream terraces. Four fields were located in the Mississippi Delta Region in the Southern Mississippi River Alluvium Area (MLRA 131A), where artificial drainage is typical and thick deposits of sandy to clayey alluvium were deposited by rivers [33].

### 2.2. Spectral and Laboratory Data Collection 

**Figure 1:**
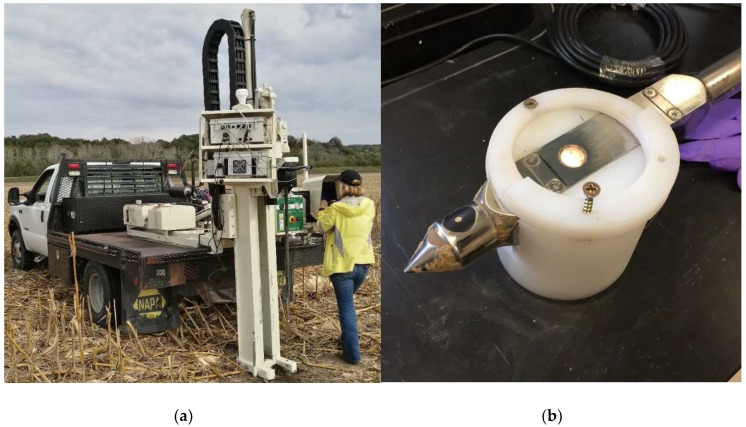
The Veris P4000 VIS-NIR instrument (**a**) and close-up view of the P4000 probe tip adapted for the laboratory (**b**).

In situ DRS soil profile spectral data were collected at 153 locations within the 22 fields described above, to a depth of ca. 1 m using a Veris P4000 (Figure 1a). The probe acquired VIS-NIR data through a sapphire window (43–367, Edmund Optics, Barrington, NJ, USA). The P4000 used a Si charge-coupled device array spectrometer and an InGaAs photodiode-array spectrometer to collect visible and near-infrared measurements in the range of 343 to 2202 nm. Dark current and reflectance standard calibrations were performed according to the manufacturer’s recommendations [27]. P4000 VIS-NIR absorbance (i.e., log_10_[1/reflectance]) measurements were obtained at a nominal 20 Hz rate as the probe was hydraulically pushed into the soil at approximately 30 mm s^−1^. To increase the signal-to-noise ratio, output data representing the mean of every 25 raw measurements were obtained at approximately 4 cm depth increments to at least 90 cm depth.

Soil cores were collected at the same locations as the probe data, split by horizon, air-dried, and sieved (<2 mm) for a total of 708 samples. Soil samples were analyzed for SOC and TN with a Leco TruMac C/N combustion analyzer (LECO Corp., St. Joseph, MI, USA) following standard procedures [34]. Water content was determined gravimetrically by oven drying, and soil texture fractions (clay, silt, and sand) were determined by the sieve-pipette method [35]. DRS spectra were also collected on the air-dried samples using the Veris P4000 (adapted for the laboratory; Figure 1b). To improve the signal-to-noise ratio, spectral values from 343–500 nm were removed. 

### 2.3. Alignment of Profile Spectra and Laboratory Data

As soil and sensor data were collected at different depth increments, it was necessary to combine them to a common level of spatial (i.e., vertical) support. This was done using weighted averaging of the sensor data to match the soil samples segmented by variable thickness horizons from the soil cores. The weighting procedure was based on the fact that the sensor depth recorded was the final depth of the instrument at the end of the 25-scan observation period. This depth then defined the starting depth for the next observation in the probing sequence. These sensor-data depth segments varied somewhat in thickness, with an average thickness of 4.0 cm and a standard deviation of 1.3 cm. The initial starting depth for the first observation in any probe was unknown; therefore, we chose to start at a depth of zero for the first scan, or at a depth such that the first observation represented no more than 4.0 cm of depth. Observations that fell entirely into a single target soil core layer were weighted by the depth increment of the observation divided by the total thickness of the layer. Where observations spanned two soil layers, the observation was partitioned into both layers based on the amount of depth represented in each layer and again divided by the layer thickness. At the end of this procedure, the weighted average sensor data were merged with the corresponding soil properties. Observations with any missing laboratory or spectral data were dropped, resulting in a final dataset of 708 observations for analysis. This dataset was further split into three sets via random sampling for validation purposes. The sample sizes for the model training, model testing, and EPO calibration datasets were 308, 200, and 200, respectively. Descriptive statistics, including the maximum, minimum, mean, and standard deviation of soil properties for each independent dataset, can be found in Table 2. Finally, a complete set of analyses were run ten additional times with different random model training, model testing, and EPO calibration datasets, and results were similar to those presented. This indicates that the conclusions were not sensitive to the randomization procedure.

### 2.4. External Parameter Orthogonalization (EPO)

External parameter orthogonalization (EPO) was applied to an independent dataset consisting of field moist and dry scans (n = 200) to decompose the spectra into a useful signal component and a nonsignal component attributable to external factors, as described in [9] and [16]. In this case, the external factor was moisture content, and the goal was to remove this component, effectively isolating the signal. This was accomplished via a linear transformation, which was then applied to the training and testing datasets as a pre-processing step prior to model development for prediction of soil properties. The EPO algorithm contains the following steps:

1. Standardize both the field moist spectra and the dry spectra to have mean zero and unit standard deviation for each soil sample. Note that for a dataset with rows corresponding to soil samples and columns corresponding to wavelengths, this step is completed via row standardization.
2. Let matrix **D** be the difference between the field moist spectra and dry spectra.
3. Perform a singular value decomposition on $D^{\prime }D$ to obtain $U\Sigma V^{\prime }$. Here, **U** denotes the matrix of left singular vectors, **V** denotes the matrix of right singular vectors, and **Σ** denotes the diagonal matrix of non-negative singular values.
4. Let matrix $Q\;=\;V_{K}\;V_{K}^{\prime }$, where $V_{K}$ consists of the first K right singular vectors of **V**.
5. The EPO transformation matrix is defined as **P = I − Q.**

Here, K is a tunable parameter that represents the number of EPO factors on which to orthogonalize. Using the transformation matrix, the EPO-transformed spectra can be found by X^*^ **= XP**, where **X** is the untransformed spectra. See [9] for further details.

### 2.5. Statistical Models 

Seven different analyses were implemented using the training set (n = 308) to fit the model and the testing set (n = 200) to calculate the out-of-sample root mean square error of prediction (RMSEP). In models where EPO was applied, the independent EPO dataset was used to create the EPO projection matrix, which was then applied to the training and testing spectral datasets. First, PLS regression models were fit as follows: (1) trained and tested on dry spectra, (2) trained on dry spectra and tested on field moist spectra, and (3) trained and tested on field moist spectra. Next, a PLS model was fit to EPO-transformed dry spectra and tested on EPO-transformed field moist spectra, then fit to EPO-transformed field moist spectra and tested on EPO-transformed field moist spectra. For all PLS models, a 10-fold cross validation was used to select the number of PLS components to retain using the one-standard-error heuristic [36] as the retention criterion to determine the optimum number of components. Specifically, models were fit with 1–50 PLS components and then the model that minimized the cross-validation error was found. The model with the smallest number of components such that the RMSEP was within one standard error of the minimum was retained. All PLS work was conducted using the pls package in R [37].

The final two model types utilized were Bayesian hierarchical models in the form of the Bayesian Lasso [38]. The Bayesian Lasso provides a form of regularization that shrinks the regression coefficient values towards zero. Regularization introduces additional information to prevent overfitting. This adds bias to the predictions, but can often reduce the variance to a greater extent, thus reducing the overall mean squared error (MSE). The shrinkage proceeds by assuming a double exponential prior distribution on the coefficients. The full model hierarchy used is as follows:

$$\;\tilde{y}|X,\;\beta ,\sigma^{2}∼N_{n}\left(X\beta ,\;\sigma^{2}I_{n}\right)\;$$

$$\beta |\;\tau_{1}^{2},\ldots ,\tau_{p}^{2},\sigma^{2}∼N_{p}\left(0_{p},\;\sigma^{2}D_{\tau }\right),\;D_{\tau }=diag\left(\tau_{1}^{2},\ldots ,\tau_{p}^{2}\right)\;$$

$$\;\tau_{1}^{2},\ldots ,\tau_{p}^{2}∼\overset{p}{\underset{j=1}{\prod }}\frac{\lambda^{2}}{2}\;\exp\left(\frac{\lambda^{2}\tau_{j}^{2}}{2}\right)\tau_{j}^{2},\;\tau_{1}^{2},\ldots ,\tau_{p}^{2}>0\;$$

$$\;\lambda^{2}∼Gamma\left(r,\delta \right)\;$$

$$\;\sigma^{2}∼\frac{1}{\sigma^{2}}\;$$

In this model, $\tilde{y}$ represents the dependent variable after centering to have mean zero. The independent variables are represented with X and should be standardized to have mean zero and unit standard deviation. The parameters r and δ can be chosen to be weakly informative, essentially letting the data outweigh the prior distribution so that these parameters impart little impact on the analysis. In this case, the value 0.1 was used for both r and δ. This model hierarchy is fully conjugate and allows for the use of Gibbs sampling to sample from the joint posterior distribution of the parameters (see Appendix A or [38] for further details). Because of the posterior distribution of the parameters, predictions are averaged over the values in the posterior sample and therefore constitutes a type of Bayesian model averaging (see [39]).

For the Bayesian Lasso models, the first 50 PLS components of the EPO-transformed spectra served as the independent variables. Each model was trained on the EPO-transformed dry spectra and tested on the EPO-transformed field moist spectra. When fitting the Bayesian Lasso models, the model was fit on the logit-transformed response for sand, silt, and clay content, given that these responses are proportions and bounded between 0 and 1. Working on the logit-transformed scale puts the response on the real line and thus makes the normally distributed response assumption of the Bayesian Lasso more appropriate. Predictions were then transformed back to the original scale, thus the interpretation of RMSEP was not affected (that is, the calculated RMSEP corresponds to the original scale). In some cases, the Bayesian Lasso may not provide enough shrinkage for unimportant covariates. For this reason, a prefilter step was implemented for the Bayesian Lasso models. The number of PLS components was varied from 2–50 for all models, and the one with minimum RMSEP on the test set was selected. The final model type added a categorical independent variable to the Bayesian Lasso model that classified each sampling location according to Loam, Sand, or Clay soil type based on the dominant type in each field (Loam (loam, silt loam), Sand (sandy loam), or Clay (claypan silt loam, clay, silty clay loam)). Note that all Bayesian Lasso models ran for 1600 iterations, discarding the first 100 iterations as burn-in. Convergence was extremely rapid and was assessed through visual inspection of the trace plots of the sample chains of the parameters, with no lack of convergence detected.

To select the optimal model within each model type, RMSEP was compared across many tuning parameter combinations. For the EPO-based models relying only on the PLS package, the number of PLS components selected by the PLS package for each level of EPO factors from 1–10 was determined. In this way, the best model was selected for each level of EPO factors, and subsequently, the best overall model was selected from among all EPO levels. A similar approach was used for the Bayesian Lasso models. Each combination of EPO factors (from 1–10) and prefiltered covariates (from 2–50 PLS factors) was evaluated. From these combinations, the best model for each soil property was selected. 

## 3. Results and Discussion

**Figure 2:**
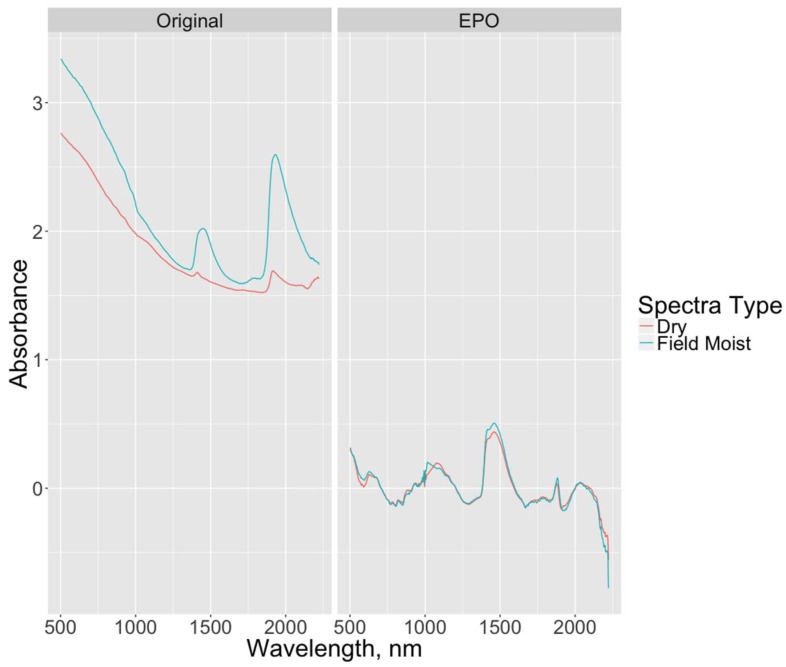
Plots of spectra before and after EPO transformation with six EPO factors for a selected soil sample. Differences in spectral features between the dry and field moist spectra are significantly reduced as a result of the transformation.

**Figure 3:**
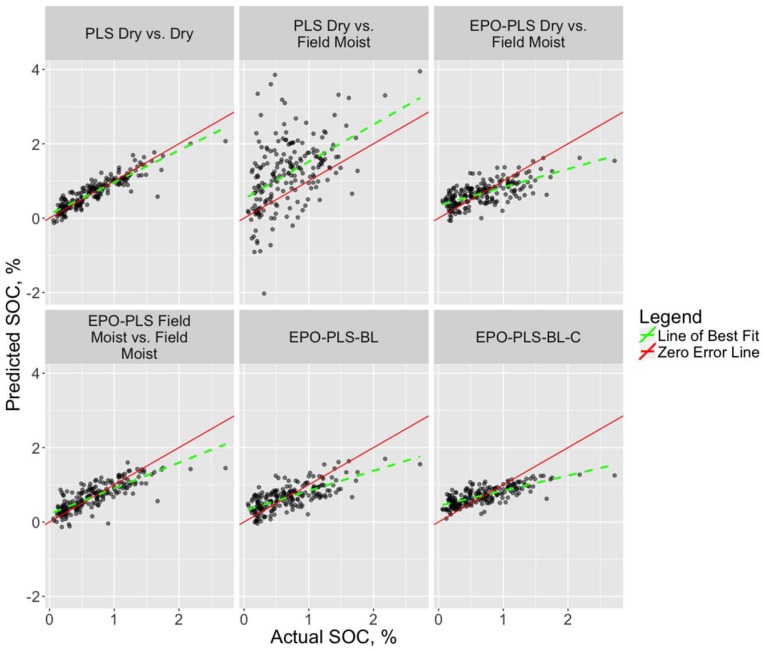
Scatterplots of actual and predicted values for the validation (test) dataset with soil organic carbon as the response. The red line represents the 1:1 (actual = predicted). For a perfect model, all points would fall on this line (zero-error line). The dashed green line represents the line of best fit for the points.

**Figure 4:**
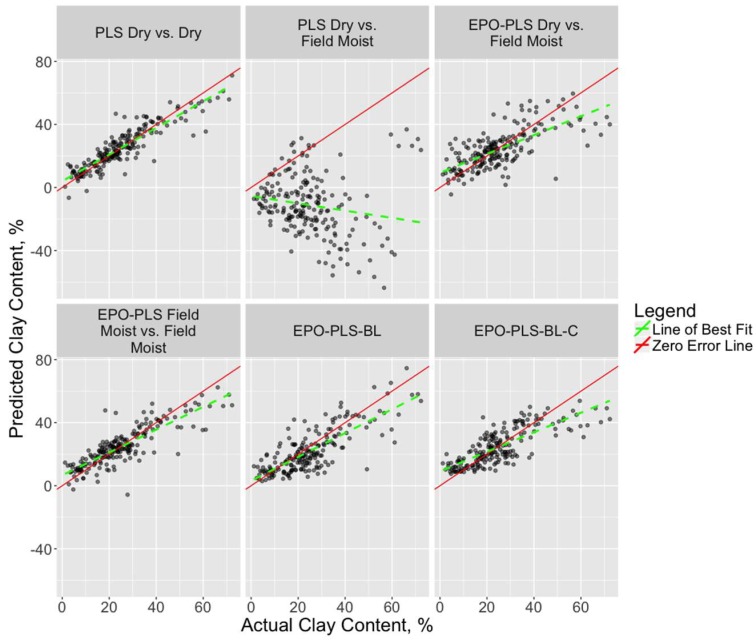
Scatterplots of actual and predicted values for the validation (test) dataset with clay content as the response. The red line represents the 1:1 (actual = predicted). For a perfect model, all points would fall on this line (zero-error line). The dashed green line represents the line of best fit for the points.

The spectral effects of EPO transformation are evident in Figure 2. The absorbance features of the field moist and dry spectra are strikingly different prior to the EPO transformation due to the effects of soil moisture. Following transformation of the spectra via EPO with six factors, the curves visibly match each other quite well in this example, indicating that the EPO transformation worked as intended. Table 3 presents the RMSEP for the best model across all model types for each soil property, along with the number of EPO and PLS factors. The number of PLS factors reflects the number chosen by the PLS package for PLS and PLS-EPO models, or the number kept in the prefilter step for the Bayesian Lasso models. The R^2^, bias, and slope values refer to the best-fit line of the actual versus predicted values for each model type and soil property, although model R^2^ was not used as a model selection criterion in this study. Scatterplots of actual versus predicted values of SOC and clay content for select models are shown in Figure 3 and Figure 4, respectively, along with the 1:1 (actual = predicted) line (zero-error line) and the best-fit line.

### 3.1. PLS Models

As expected, the PLS models with the dry training set and dry testing set performed the best across all soil properties, as indicated by the smallest RMSEP for SOC, TN, and the texture fractions (R^2^ ranging from 0.68 to 0.82). PLS models with the field moist training and test sets demonstrated somewhat lower performance, with a 7–41% increase in RMSEP relative to the PLS models trained and tested on dry spectra. In contrast, the RMSEP for PLS models trained on dry spectra and tested on field moist spectra was very large compared to results trained and tested on dry spectra, reflecting a 3- and 4-fold increase for TN and SOC, respectively, and a 6-, 13-, and 3-fold increase for clay, silt, and sand, respectively (R^2^ ranging from 0.03 to 0.23). These results are consistent with previous work showing a reduction in performance when calibrating with dry spectra and predicting with moist spectra [5,15]. Despite the reduction in performance, there are potential advantages of training models on dry spectra for prediction with field moist spectra, namely that soil samples collected for standard laboratory analyses, such as SOC and TN, are typically processed by drying and sieving. Thus, dry spectra could readily be collected on these processed samples to generate a training dataset. Subsequently, these dry calibration models could be used to predict soil properties with dry spectra collected in a lab or with field moist spectra collected at higher spatial resolution under variable conditions. However, in this study, PLS models trained on dry spectra did not perform well when predicting soil properties with field moist spectra. Thus, the goal of leveraging existing libraries of spectra collected from dry, processed soil to predict soil properties using field moist spectra was not realized using only PLS on untransformed spectra from this regional dataset, and alternative techniques were explored.

### 3.2. EPO-PLS Models

The EPO transformation of the spectra provided substantial reduction in RMSEP for each soil property for models trained on dry spectra and tested on field moist spectra, demonstrating RMSEP reductions from 53–91% across soil properties. This improvement is evident in the scatterplots shown in Figure 3 and Figure 4, where the best-fit and zero-error lines are either widely separated (SOC) or divergent (clay content) with the PLS-only models (R^2^ = 0.23 and 0.03, respectively), whereas the EPO-PLS models greatly improve the relationship between the actual and predicted values (R^2^ = 0.46 and 0.49, respectively). 

The advantage of robust models that are trained on dry spectra for prediction with field moist spectra lies in the ability to develop spectral libraries consisting of soil properties and EPO-transformed dry spectra collected in the laboratory. Subsequently, in situ profile spectra could be collected under variable environmental conditions at new locations and at high spatial resolution, the EPO transformation could be applied to these field moist spectra, and predictions of multiple soil properties could be obtained without the cost of soil core collection or laboratory analysis.

### 3.3. EPO-PLS-Bayesian Lasso Models and Covariate Addition

The application of the Bayesian Lasso to the EPO-PLS components further improved performance for models trained on dry spectra and tested on field moist spectra. For SOC and TN, the EPO-PLS-BL model reduced RMSEP by 4% and 9%, respectively, over the EPO-PLS models. For clay, silt, and sand, RMSEP was reduced by 10%, 4%, and 11%, respectively, relative to the EPO-PLS models. The addition of the soil classification covariate to the Bayesian Lasso models (EPO-PLS-BL-C models) demonstrated a reduction of RMSEP by 5–20% over the EPO-PLS models, with strong improvements observed in the clay, silt, and sand fractions.

**Figure 5:**
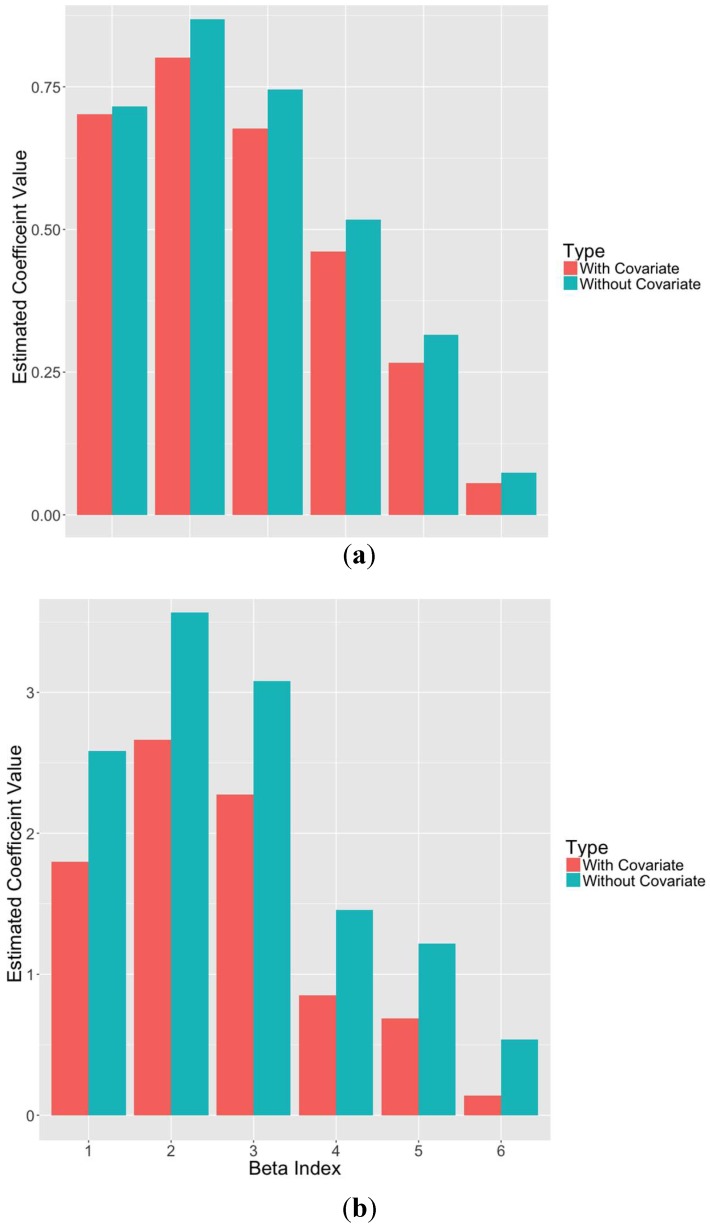
Estimated coefficient values for Bayesian Lasso models with and without the soil type covariate for SOC (**a**) and sand content (**b**). Please refer to Table 3 for the number of EPO and PLS factors.

In general, one strength of the Bayesian Lasso lies in the addition of covariates to the model. Components in PLS models are designed to be uncorrelated, but additional covariates will likely exhibit some level of correlation with the PLS components. The Bayesian Lasso, or other forms of regularization, can reduce the prediction variance and in turn reduce mean squared error (MSE). Figure 5 illustrates the reduction of the coefficient estimates for the Bayesian Lasso model with and without the added covariate for prediction of SOC and clay content. In this example, the first six PLS components were used with 10 EPO factors. This reduction indicates that information contained in the covariate is already contained in the PLS factors, and thus the inputs are correlated to some extent. To combat this multicollinearity, the Bayesian Lasso shrinks the coefficients towards zero. In this case, the observed improvement in the EPO-PLS-BL-C models for clay, silt, and sand was expected and is intuitive, given that soil texture is a diagnostic characteristic of soil taxonomy and classification. Thus, in this case, the covariate assisted in model performance by providing a useful intercept or starting point for prediction of clay, silt, and sand content.

## 4. Conclusions and Future Work 

This study demonstrated the potential for in situ profile DRS spectral data to predict soil properties under variable field conditions, using the EPO transformation in conjunction with the Bayesian Lasso along with additional covariate information using models developed on dry spectra. The main benefit of this approach lies in the ability to leverage existing libraries of spectra and soil properties measured in the laboratory on dry, processed soil samples to develop PLS-EPO-BL calibration models. Alternatively, soil samples handled and processed for standard laboratory analyses or archived soil samples could be used as training sets to develop PLS-EPO-BL calibration models. Subsequently, these models could be used to predict soil properties on EPO-transformed field moist spectra collected at new locations and at high resolution without the expense of soil collection and analysis.

Future work in this area involves the evaluation of additional statistical approaches in combination with techniques such as sensor data fusion. Given that the strength of the Bayesian Lasso lies in the ability to add covariates, additional variables should be considered in future studies. Further, it is also possible to use the Bayesian Lasso on the spectra directly without PLS projections. This approach could result in increased computation time, although it has the advantage of increased interpretability and a potential reduction in RMSEP. In this case, Bayesian coefficient values may be used to gain insight into the importance of spectral features in prediction of soil properties. This is not possible in many cases under ordinary least squares regression, due to the number of wavelengths sampled in the spectrum being larger than the sample size used to fit the model. Ultimately, there are many opportunities for continued work to unlock the potential of profile DRS spectroscopy under different field conditions, including making the procedure fully automated. The ability to develop a spectral library with regional calibration models built on EPO-transformed dry spectra that can successfully predict soil properties using in situ field moist spectra would be beneficial for site-specific precision agriculture, soil health assessment, and many other applications.

## Figures and Tables

**Table 1:** Profile locations and soil descriptions of sampling locations in Missouri and Indiana by major land resource area (MLRA). In situ scans and soil cores were collected from each location (n = 153). Cores were split by horizon, resulting in 708 total samples.

Location	Soil Textural Class	Taxonomic Class	# Fields	# Profiles
Indiana Outwash MLRA 98	Loam; Sandy loam	Sebewa loam: Fine-loamy over sandy or sandy-skeletal, mixed, superactive, mesic Typic Argiaquolls; Tracy sandy loam: Coarse-loamy, mixed, active, mesic Ultic Hapludalfs	6	24
Central Missouri Claypan MLRA 113	Silt loam	Adco silt loam: Fine, smectitic, mesic Vertic Albaqualfs; Mexico silt loam: Fine, smectitic, mesic Vertic Epiaqualfs; Leonard silt loam: Fine, smectitic, mesic Vertic Epiaqualfs	6	60
Missouri Upland Loess MLRA 109	Silt loam; Silty clay loam	Higginsville silt loam: Fine-silty, mixed, superactive, mesic Aquic Argiudolls; Wakenda silt loam: Fine-silty, mixed, superactive, mesic Typic Argiudolls; Knox silty clay loam: Fine-silty, mixed, superactive, mesic Mollic Hapludalfs	3	23
Missouri River Alluvium MLRA 115B	Silt loam; Silty clay loam	Lowmo silt loam: Coarse-silty, mixed, superactive, mesic Fluventic Hapludolls; Peers silty clay loam: Fine-silty, mixed, superactive, mesic Fluvaquentic Hapludolls	3	12
Mississippi River Delta Alluvium MLRA 131A	Clay; Sandy loam; Loam, Silt loam;	Tiptonville silt loam: Fine-silty, mixed, superactive, thermic Oxyaquic Argiudolls; Reelfoot loam and sandy loam: Fine-silty, mixed, superactive, thermic Aquic Argiudolls; Steele sandy loam: Sandy over clayey, mixed, superactive, nonacid, thermic Aquic Udifluvents; Dundee silt loam: Fine-silty, mixed, active, thermic Typic Endoaqualfs; Portageville clay: Fine, smectitic, calcareous, thermic Vertic Endoaquolls; Dubbs silt loam: Fine-silty, mixed, active, thermic Typic Hapludalfs	4	34

**Table 2:** Maximum, minimum, mean, and standard deviation (SD) of laboratory-determined soil properties for the training, testing, and external parameter orthogonalization (EPO) calibration datasets. All units are in % (g × 100 g soil^−1^).

	Training (n = 308)				Testing (n = 200)				EPO Calibration (n = 200)			
	Max	Min	Mean	SD	Max	Min	Mean	SD	Max	Min	Mean	SD
SOC ^†^	2.95	0.06	0.70	0.45	2.72	0.06	0.68	0.44	1.98	0.03	0.65	0.43
TN ^‡^	0.23	0.01	0.06	0.04	0.21	0.01	0.06	0.04	0.16	0.01	0.06	0.04
Sand	98.0	0.6	22.1	26.0	96.2	0.5	24.2	27.2	97.8	0.3	23.7	28.6
Silt	83.7	1.2	51.4	18.7	81.9	2.6	50.8	19.8	81.3	1.4	49.9	20.0
Clay	68.9	0.8	26.4	14.4	72.3	1.2	25.0	14.5	69.7	0.8	26.4	15.5
Moisture	41.8	2.8	23.3	6.4	73.9	3.8	22.5	7.6	42.2	4.6	22.7	6.7

^†^ SOC = soil organic carbon; ^‡^ TN = total nitrogen.

**Table 3:** The root mean square error of prediction (RMSEP) for the best model within each model type including partial least squares (PLS), external parameter orthogonalization (EPO) transformation, Bayesian Lasso (BL), and covariate addition (C) for each soil property: soil organic carbon (SOC), total nitrogen (TN), and particle size fractions in % (g × 100 g^−1^ soil). The EPO transformation was determined on an independent set of field moist and dry spectra (n = 200), and the number of EPO factors is shown. The number of PLS factors corresponds to the number chosen by the PLS package in PLS and PLS-EPO models, and refers to the number kept in the prefilter step for Bayesian Lasso models. R^2^, bias, and slope represent the best-fit line between the actual and predicted values for each soil property and model type.

Soil Property	Model Type	Training Set (n = 308)	Test Set (n = 200)	# PLS Factors	# EPO Factors	RMSEP	R^2^	Bias	Slope
**SOC**	PLS	Dry	Dry	14	0	0.188	0.82	0.01	0.86
**SOC**	PLS	Dry	Field Moist	14	0	0.960	0.23	0.52	1.00
**SOC**	PLS	Field Moist	Field Moist	14	0	0.265	0.64	−0.01	0.69
**SOC**	EPO-PLS	Dry	Field Moist	12	6	0.327	0.46	−0.01	0.49
**SOC**	EPO-PLS	Field Moist	Field Moist	9	7	0.262	0.65	0.01	0.69
**SOC**	EPO-PLS-BL	Dry	Field Moist	13	6	0.316	0.49	−0.01	0.54
**SOC**	EPO-PLS-BL-C	Dry	Field Moist	3	5	0.310	0.55	0.03	0.41
**TN**	PLS	Dry	Dry	13	0	0.017	0.81	0.00	0.81
**TN**	PLS	Dry	Field Moist	14	0	0.068	0.20	0.02	0.81
**TN**	PLS	Field Moist	Field Moist	12	0	0.024	0.63	0.00	0.67
**TN**	EPO-PLS	Dry	Field Moist	10	6	0.032	0.34	0.00	0.43
**TN**	EPO-PLS	Field Moist	Field Moist	8	6	0.024	0.63	0.00	0.68
**TN**	EPO-PLS-BL	Dry	Field Moist	4	3	0.029	0.52	0.00	0.34
**TN**	EPO-PLS-BL-C	Dry	Field Moist	3	5	0.027	0.53	0.00	0.44
**Clay**	PLS	Dry	Dry	11	0	6.281	0.81	0.11	0.84
**Clay**	PLS	Dry	Field Moist	11	0	44.539	0.03	−36.26	−0.23
**Clay**	PLS	Field Moist	Field Moist	11	0	8.388	0.66	−0.61	0.69
**Clay**	EPO-PLS	Dry	Field Moist	12	9	10.597	0.49	−0.73	0.60
**Clay**	EPO-PLS	Field Moist	Field Moist	8	6	7.775	0.71	−0.28	0.72
**Clay**	EPO-PLS-BL	Dry	Field Moist	16	8	9.594	0.63	−2.98	0.76
**Clay**	EPO-PLS-BL-C	Dry	Field Moist	3	10	9.048	0.61	−0.38	0.62
**Silt**	PLS	Dry	Dry	14	0	11.214	0.68	0.30	0.69
**Silt**	PLS	Dry	Field Moist	14	0	159.498	0.08	−156.88	0.79
**Silt**	PLS	Field Moist	Field Moist	13	0	11.964	0.63	−0.40	0.64
**Silt**	EPO-PLS	Dry	Field Moist	6	10	15.013	0.42	−0.97	0.43
**Silt**	EPO-PLS	Field Moist	Field Moist	12	1	11.908	0.63	−0.25	0.65
**Silt**	EPO-PLS-BL	Dry	Field Moist	5	8	14.433	0.47	−1.39	0.46
**Silt**	EPO-PLS-BL-C	Dry	Field Moist	5	8	13.496	0.53	−0.30	0.56
**Sand**	PLS	Dry	Dry	18	0	13.081	0.77	−0.08	0.85
**Sand**	PLS	Dry	Field Moist	17	0	155.874	0.23	−79.94	2.04
**Sand**	PLS	Field Moist	Field Moist	13	0	12.069	0.75	0.59	0.75
**Sand**	EPO-PLS	Dry	Field Moist	9	10	19.899	0.54	0.25	0.74
**Sand**	EPO-PLS	Field Moist	Field Moist	15	1	14.289	0.72	0.48	0.73
**Sand**	EPO-PLS-BL	Dry	Field Moist	6	10	17.855	0.58	−1.21	0.68
**Sand**	EPO-PLS-BL-C	Dry	Field Moist	4	10	16.197	0.66	−2.82	0.63

## Appendix A

The full conditional distributions that we used to implement the Bayesian Lasso Gibbs sampler can be found below (see [40] for details on Gibbs sampling). Specifically, one can sample iteratively from the list of full conditionals in order to sample from the joint posterior distribution of the parameters. The full conditionals are as follows:

$$\beta |\cdot ∼\;N_{p}\left(A^{-1}X^{\prime }\tilde{y},\;\sigma^{2}A^{-1}\right),A^{-1}=X^{\prime }X+D_{\tau }^{-1}$$

$$\sigma^{2}|\cdot ∼\;IG\left(\frac{n-1}{2}+\;\frac{p}{2}+1,\;\frac{{\left(\tilde{y}-X\beta \right)}^{\prime }\left(\tilde{y}-X\beta \right)}{2}+\;\frac{\beta^{\prime }\;D_{\tau }^{-1}\beta }{2}\right)$$

$$\frac{1}{\tau_{j}^{2}}\;|\cdot ∼\;Inv.Gauss\left({\left(\frac{\lambda^{2}\sigma^{2}}{\beta_{j}^{2}}\right)}^{\frac{1}{2}},\;\lambda^{2}\right),j=1,\ldots ,\;p$$

$$\lambda^{2}|\cdot ∼\;Gamma(p+r,\;\sum_{j=1}^{p}\frac{\tau_{j}^{2}}{2}+\delta ),$$

where *Dτ* is defined in the main text. Here, *IG* (*A*,*B*) represents the Inverse Gamma distribution with shape parameter A and scale parameter B. The Inverse Gaussian distribution with mean parameter A and scale parameter B is represented by *Inv.Gauss* (*A*,*B*). Finally, *Gamma* (*A*,*B*) represents the Gamma distribution with shape parameter A and rate parameter B. See [41] for further details.

The above full conditionals assume no intercept coefficient, because the response and covariates have been mean-centered. Alternatively, one could add an intercept to the model, allowing for the use of the response data y without centering to obtain mean zero. The data level of the model then becomes:

$$y|X,\;\beta ,\sigma^{2}∼N_{n}\left(\theta_{n}+X\beta ,\;\sigma^{2}I_{n}\right)$$

Here, $\theta_{n}$ just represents a vector of length *n* with all values equal to θ. The full conditional for the intercept parameter is as follows:

$$\theta |\cdot ∼\;N\left(\bar{y}-\bar{X}\prime \beta ,\;\frac{\sigma^{2}}{n}\right).$$

When including an intercept in the model, the full conditionals for β and $\sigma^{2}$ take the following form:

$$\beta |\cdot ∼\;N_{p}\left(A^{-1}X^{\prime }\left(y-\theta_{n}\right),\;\sigma^{2}A^{-1}\right)$$

$$\sigma^{2}|\cdot ∼\;IG\left(\frac{n-1}{2}+\;\frac{p}{2}+1,\;\frac{{\left(y-\theta_{n}-X\beta \right)}^{\prime }\left(y-\theta_{n}-X\beta \right)}{2}+\;\frac{\beta^{\prime }\;D_{\tau }^{-1}\beta }{2}\right).$$

The full conditionals for all other parameters do not differ from the case without an intercept.

In our analysis, we include an intercept term for the models without the soil type covariate, but remove the intercept when the additional covariate is present.
